# Hospitals and the War

**Published:** 1915-01-02

**Authors:** 


### HOSPITALS AND THE WAR.

**(From our Special Correspondents.)**

### CARDIFF.

On Sunday, December 27, at 4.30 a.m., there arrived at Cardiff from Southampton another 180 soldiers. They were met at the Great Western railway station by Major E. Maclean and a large staff of ambulance men, and amid pouring rain were conveyed with all speed to the various hospitals. There were no serious cases, and the majority of the men will be able to return to the Front shortly. The men were distributed as follows: seventy to Albany Road, twenty to King Edward VII.'s Hospital, sixty to Splott Road, and thirty to Howard Gardens.

Since the opening of the 3rd Western General Hospital 1,800 patients have been admitted from overseas and 350 home troops. The total admissions have been 2,150 and the discharges about 2,000. Only three soldiers have died, and it is a matter for public interest that cases requiring careful investigation have been expressly sent over to Cardiff from France.

### LEICESTER: A CHRISTMAS CONVOY.

On the evening of Boxing Day a message received from Southampton announced the dispatch of a train-load of sick and wounded to the Leicester Base Hospital, and prompt arrangements were made by the County Director Voluntary Aid Detachments, Mr. A. W. Faire, for assembling nurses and St. John Ambulance attendants to meet the train, and a number of ambulances and motor-cars for transport. At holiday-times these arrangements are by no means easy of accomplishment, and it was therefore a matter of disappointment that the expected convoy did not arrive at the time expected. After a long wait, however, a message was received that the train would arrive about six o'clock on Sunday morning, and there was nothing to be done but to wait through the night for the delayed train. The men, who had spent Christmas Day at Boulogne, where they seem to have been very well looked after, consisted principally of medical cases, mostly slight cases of frostbite, rheumatism, etc., had had an exceedingly rough passage to Southampton, and they were therefore very glad of the rest the transport train afforded, and in less than half an hour the last patient had been borne to a waiting motor and was on his way to the hospital, where Lieut.-Col. Harrison had made every provision for care and reception of the new arrivals. On Saturday evening an excellent entertainment, arranged by the ladies' committee, was given at the base hospital. Lieut.-Col. Harrison was in the chair, and the Mayor and Mayoress, Alderman and Mrs. North, were present with many of the members of the hospital staff and their wives. By the co-operation of "Ford" motorists, a Ford ambulance waggon has been added to the equipment of the local Voluntary Aid Detachment for motor-transport, and will be a source of much help to the County Director.

### NETLEY: THE WELSH WAR HOSPITAL.

THE Welsh Hospital at Netley, under Colonel Sheen, has settled steadily down to work. The weather has been trying of late, but discomfort is minimised by the open-floored corridor connecting the different portions. The semi-open-air life seems to contribute greatly to the health of the staff, all of whom are exceedingly well. Over the majority of the beds in the wards is a label indicating the source from which the bed was endowed, and these cause great interest and amusement to the "Tommies." The "Lloyd-George" bed, the "Suffragist" bed, and the "Welsh Dogs'" bed are the subject of much comment. Extensions are being rapidly made, a room for sick officers, recreation room for patients, and a nursing sisters' sitting room being amongst others which will add to the comforts of the hospital. Concerts are held weekly or oftener in one of the wards.

The majority of the wounds created have been by shrapnel and "coal-boxes." Many of the injuries are to the arms, owing to the men being in the trenches when hit. Gifts have been many, and the soldiers are bearing their troubles with fortitude.

### NORFOLK AND NORWICH HOSPITAL.

THE eighth convoy of wounded and sick soldiers from Boulogne arrived at this hospital on the evening of December 23, making a total of 800 since the outbreak of war. One hundred and sixty soldiers were in the institution on Christmas Day, but no elaborate decorations were made in the hospital to celebrate it, nevertheless pains were taken that every patient might find himself in a Christmas atmosphere. Every man received a Christmas card from the King and Queen and a box of cigarettes from Queen Alexandra. Miss Larking presented every man with a soldier's companion stocked with stationery, and her father (the chairman of the board of management) gave them each an inscribed case containing a cigar and cigarettes; he also sent a similar gift to all the old patients of the hospital who were in convalescent homes. One of the recipients, on taking out his cigar, solemnly uttered a promise that he would not smoke it until peace was declared. Concerts were given by Mrs. Beeching and Mrs. Blaxland. It may be interesting to note that in one of the tent wards there is a patient (Driver Baker, R.F.A.) who has been recommended for the Victoria Cross, whilst another (Sergeant Alexander Stuart, of the 2nd Yorkshire Regiment) has won the Distinguished Conduct Medal. The new tent for the soldiers is progressing as far as the unfavourable weather will permit, and is expected to be opened early in February. The cost of this building will be defrayed by the *Eastern Daily Press* Fund.

### INDIANS AT BRIGHTON.

THE base hospital which is in process of being established at Brighton for men of the Indian branch of the Expeditionary Force will contain about three thousand beds located partly in the Dome, Pavilion, and Corn Exchange, placed at the disposal of the Government by the Corporation of Brighton, partly in York Place School, and partly in the Poor-Law institution, the Guardians of the Poor having undertaken to remove the fifteen hundred inmates at as early a date as possible.

The hospital is being staffed mainly by retired Indian Medical Service men, partly by officers of the Royal Army Medical Corps, and partly by Indian native doctors. Lieutenant-Colonel Neil Campbell, a well-known Anglo-Indian officer, is in supreme command, with Lieutenant-Colonel Elliot as registrar. Other well-known members of the Service are acting as surgeons, etc.

There are a good many matters in organising this hospital which require different treatment from those we are accustomed to in Great Britain, notably the food arrangements and sanitary matters. In the latter example, the baths are all shower-baths, as the native does not immerse himself as we do, and the closets are differently arranged. The preparation of the food is carried out in two different sets of kitchens with different cooks and utensils, one for the Hindus together with the Sikhs and Gurkhas, and the other for the Mussulmans. The chief articles of diet consumed are curry, chupattis, and rice. The language difficulty is not so great as it seems, for, of course, the members of the Indian Medical Service speak the dialects, and there are a good many native doctors and senior students to assist.

The buildings, although not complete, are so well advanced that already some three hundred patients have been received. Each of the three branches of the hospital—the Pavilion branch, the York Place branch, and the workhouse branch—will be quite complete in itself, with its own operating theatres, *x*-ray department, dispensary, stores, etc. The nursing arrangements are in the hands of men of the R.A.M.C. and of men of the St. John Ambulance Association. They will be supervised by nursing sisters, who will also have the care of the operating theatres, but will take no part in the actual nursing details.

### MIDDLESBROUGH: A MEMORY OF THE SCARBOROUGH RAID.

THIS hospital had its first experience of the effects of naval warfare on Wednesday, December 16. On that day H.M.S. *Patrol*, badly damaged in the raid on the North-East Coast, was able to make its way up the Tees to Middlesbrough, where it is now laid up for repairs. The cruiser brought seven wounded men, two of whom unfortunately died on the way, and the other five were brought to the hospital. It is surprising, considering the violence of the bombardment, that in a crew of more than 300 the casualties were not greater. The injuries consisted chiefly of compound fractures and serious flesh wounds. One man died within a few hours of admission; the others are now doing well. All are contented and uncomplaining. One relates how he was "knocked over" by the first shot from the German vessels, and he is happy in the knowledge that he is the first British sailor to be wounded, in the present war, in defending his country from bombardment.

Apropos of "Hospital Poetry" in a recent issue of THE HOSPITAL, it may be of interest to record the fact that "Bravo Bristol and other Verses," a little shilling volume by Fred L. Weatherley, is being sold for the benefit of the funds of the Bristol Branch of the Red Cross Society.

